# Supplementary material for: Association between Thyroid Cancer and Breast Cancer: Two Longitudinal Follow-Up Studies Using a National Health Screening Cohort
Source: J Pers Med. 2022 Jan 20;12(2):133. doi: 10.3390/jpm12020133 (PMC8880453; doi:10.3390/jpm12020133)
Supplement: Supplementary file 1 [file jpm-12-00133-s001.zip › jpm-1552153-SI.pdf]

Supplement Table S1. Detailed General Characteristics of Participants

Characteristics	Study I			Study II		
	Thyroid cancer (n, %)	Control I (n, %)	P-value	Breast cancer (n, %)	Control II (n, %)	P-value
Age (years old)			1.000			1.000
40-44	97 (2.5)	388 (2.5)		237 (7.2)	948 (7.2)	
45-49	641 (16.2)	2,564 (16.2)		684 (20.7)	2,736 (20.7)	
50-54	1,106 (28.0)	4,424 (28.0)		813 (24.6)	3,252 (24.6)	
55-59	870 (22.0)	3,480 (22.0)		573 (17.3)	2,292 (17.3)	
60-64	571 (14.5)	2,284 (14.5)		450 (13.6)	1,800 (13.6)	
65-69	375 (9.5)	1,500 (9.5)		272 (8.2)	1,088 (8.2)	
70-74	211 (5.3)	844 (5.3)		171 (5.2)	684 (5.2)	
75-79	60 (1.5)	240 (1.5)		90 (2.7)	360 (2.7)	
80-84	17 (0.4)	68 (0.4)		16 (0.5)	64 (0.5)	
85+	1 (0.0)	4 (0.0)		2 (0.1)	8 (0.1)	
Income			1.000			1.000
1 (lowest)	525 (13.3)	2,100 (13.3)		507 (15.3)	2,028 (15.3)	
2	501 (12.7)	2,004 (12.7)		441 (13.3)	1,764 (13.3)	
3	640 (16.2)	2,560 (16.2)		534 (16.1)	2,136 (16.1)	
4	821 (20.8)	3,284 (20.8)		629 (19.0)	2,516 (19.0)	
5 (highest)	1,462 (37.0)	5,848 (37.0)		1,197 (36.2)	4,788 (36.2)	
Region of residence			1.000			1.000
Urban	1,919 (48.6)	7,676 (48.6)		1,651 (49.9)	6,604 (49.9)	
Rural	2,030 (51.4)	8,120 (51.4)		1,657 (50.1)	6,628 (50.1)	
CCI score			<0.001*			<0.001*
0	3,861 (97.8)	15,588 (98.7)		3,165 (95.7)	13,034 (98.5)	
1	9 (0.2)	37 (0.2)		17 (0.5)	47 (0.4)	
2	12 (0.3)	39 (0.3)		10 (0.3)	28 (0.2)	

3	18 (0.5)	45 (0.3)		6 (0.2)	41 (0.3)	
≥ 4	49 (1.2)	87 (0.6)		110 (3.3)	82 (0.6)	
Obesity †			<0.001*			0.018
Underweight	53 (1.3)	333 (2.1)		275 (1.6)	54 (2.1)	
Normal	1,425 (36.1)	6,236 (39.5)		5,122 (36.5)	1,208 (38.7)	
Overweight	1,100 (27.9)	4,090 (25.9)		3,475 (26.7)	882 (26.3)	
Obese I	1,220 (30.9)	4,592 (29.1)		3,920 (31.2)	1,031 (29.6)	
Obese II	151 (3.8)	545 (3.5)		440 (4.0)	133 (3.3)	
Smoking status			<0.001*			0.015*
Nonsmoker	3,880 (98.3)	15,355 (97.2)		3,213 (97.1)	12,825 (96.9)	
Past smoker	29 (0.7)	142 (0.9)		40 (1.2)	110 (0.8)	
Current smoker	40 (1.0)	299 (1.9)		55 (1.7)	297 (2.2)	
Alcohol consumption			0.002*			0.019*
< 1 time a week	3,745 (94.8)	14,774 (93.5)		3,119 (94.3)	12,326 (93.2)	
≥ 1 time a week	204 (5.2)	1,022 (6.5)		189 (5.7)	906 (6.9)	
Breast cancer	39 (1.0)	94 (0.6)	0.007*	3,308 (100.0)	0 (0.0)	<0.001*
Thyroid cancer	3,949 (100.0)	0 (0.0)	<0.001*	84 (2.5)	181 (1.4)	<0.001*

Abbreviations: CCI, Charlson comorbidity index

* Chi-square test. Significance at P < 0.05

† Obesity (BMI, body mass index, kg/m^2) was categorized as < 18.5 (underweight), ≥ 18.5 to < 23 (normal), ≥ 23 to < 25 (overweight), ≥ 25 to < 30 (obese I), and ≥ 30 (obese II).
